# Survey data on the social, personal, and work resources associated with work engagement among knowledge workers in Malaysia amid the COVID-19 pandemic

**DOI:** 10.1016/j.dib.2021.107690

**Published:** 2021-12-08

**Authors:** Adedapo Oluwaseyi Ojo, Olawole Fawehinmi, Mohd Yusoff Yusliza

**Affiliations:** a Faculty of Management, Multimedia University, Cyberjaya 63100, Malaysia; b Faculty of Business, Economics, and Social Development, Universiti Malaysia Terengganu, Kuala Nerus 21030, Malaysia

**Keywords:** Resilience, Supervisor support, Family and friend support, Psychological empowerment, Facilitating conditions, Work engagement, COVID-19 pandemic

## Abstract

A regression analysis was conducted to assess the link between resilience, supervisor support, family and friend support, psychological empowerment, and facilitating conditions with work engagement using the Statistical Package of Social Sciences (SPSS) 26. This data was obtained from a cross-sectional survey of 259 knowledge workers in Malaysia. Specifically, this article provides data about the participants’ demographic characteristics and the descriptive data of participants’ responses. Further, the mean, standard deviation, reliability of the measured constructs, and regression analysis model summary are provided. This dataset offers suggestions to the top management in deducing ways to increase employees’ work engagement during the COVID-19 pandemic.

## Specifications Table

**Table:** 

Subject	Organizational Behavior and Human Resource Management
Specific subject area	Work Engagement
Type of data	Table
How data were acquired	Data was gathered using an online survey platform (Google forms).
Data format	Raw Analyzed Filtered
Parameters for data collection	The participants had to be identified as knowledge workers in the higher educational, engineering / IT services sector to be included in the sample.
Description of data collection	The online survey was purposefully distributed from April 1 to May 30, 2020, during Malaysia's first movement control order to curb the spread of the COVID-19 pandemic. The data was collected using Google form, and the survey link was shared with the potential respondents via email and social media platforms like Facebook and LinkedIn.
Data source location	Country: Malaysia 2° 30′ N and 112° 30′ Samples/data: Knowledge workers.
Data accessibility	Repository name: Mendeley.Data Repository: https://doi.org/10.17632/kpmftkdsvd.1
Related research article	Ojo, A. O., Fawehinmi, O., & Yusliza, M. Y. (2021). Examining the predictors of resilience and work engagement during the COVID-19 pandemic. Sustainability, 13(5), 2902.[1]

## Value of the Data

• This data provides essential information on capturing employees’ work engagement during the COVID-19 pandemic through employees’ resilience, supervisor support, family and friend support, psychological empowerment, and facilitating conditions.
• The data may be used by top management in developing ways to boost their employees’ work engagement, primarily through WFH arrangements during the COVID-19 pandemic. This may be through devising ways to increase and encourage employees’ resilience, supervisor support, family and friend support, psychological empowerment, and facilitating conditions to heighten employees’ work engagement.
• This data may be reused when investigating the moderating influence of supervisor support on the links between employees’ resilience, psychological empowerment, facilitating conditions, and work engagement.
• This data can be reused in studies investigating the moderating influence of family and friend support on the links between the employees’ resilience, psychological empowerment, facilitating conditions, and employees’ work engagement.

## 1 Data Description

The data is represented in six tables. Table 1 exhibits participants’ demographics, portraying participants’ demographic characteristics, such as gender and age, educational level, job position, job sector. Next, Table 2 presents the descriptive statistics of knowledge workers perceptions of their work engagement, resilience, supervisor support, family and friend support, psychological empowerment, and facilitating conditions during the COVID-19 pandemic. The data shows the mean and standard deviation of the items. Further, Table 3 depicts the mean, standard deviation, average variance extracted (AVE) and reliability of measured constructs. Table 4 displays the model summary, such as the coefficient of the determination (R^2^) of the model. Table 5 demonstrates the ANOVA regression of the model. The data shows the *F* value of the model. Table 6 shows the model's coefficient, such as the Beta and standard error of the measured constructs, *T* value and the significance of the exogenous variables. Lastly, Table 7 presents the discriminant validity of the constructs.

**Table 1:** Demographics of participants (*N* = 259).

Variables	Categories	Frequency	Percent
Gender	Female	107	41.3
	Male	152	58.7
Age	26–35 years	56	21.6
	36–45 years	117	45.2
	46–55 years	61	23.6
	Above 55 years	25	9.7
Marital Status	Single	43	16.6
	Married	208	80.3
	Divorced / Widowed	8	3.1
Educational Level	Diploma	19	7.3
	Bachelor / Equivalent	48	18.5
	Masters	55	21.2
	Doctorate	137	52.9
Job Position	Low Management	59	22.8
	Middle Management	112	43.2
	Top Management	88	34.0
Job Sector	Engineering Services	21	8.1
	IT Services	52	20.1
	Higher Education	186	71.8

**Table 2:** Descriptive statistics of knowledge workers perceptions of work engagement, resilience, supervisor support, family and friend support, psychological empowerment, and facilitating conditions during COVID-19 pandemic.

	Variables	Mean	Std. Dev.
	Work Engagement (α = 0.924)		
WKE1	At my work, I feel bursting with energy.	4.07	1.26
WKE2	At my job, I feel strong and vigorous.	4.19	1.20
WKE3	I am enthusiastic about my job.	4.55	1.08
WKE4	My job inspires me.	4.59	1.05
WKE5	When I get up in the morning, I feel like doing my work.	4.27	1.29
WKE6	I feel happy when I am working intensely.	4.56	1.21
WKE7	I am proud of the work that I do.	4.92	0.96
WKE8	I am immersed in my work.	4.47	1.15
WKE9	I get carried away when I am working.	4.36	1.18
	Resilience (α = 0.757)		
RES1	I tend to bounce back quickly after hard times.	4.99	1.36
RES2	I have a hard time making it through stressful events.*	3.39	1.90
RES3	It does not take me long to recover from a stressful event.	3.72	2.15
RES4	It is hard for me to quickly return back to normal when something bad happens.*	5.10	1.33
RES5	I usually come through difficult times with little trouble.	4.63	1.51
RES6	I tend to take a long time to get over set-backs in my life.*	3.76	2.14
	Supervisor Support (α = 0.636)		
SST1	My supervisor cares about my opinions	5.32	1.46
SST2	My work supervisor really cares about my well-being.	5.25	1.49
SST3	My supervisor strongly considers my goals and values	5.13	1.45
SST4	My supervisor shows very little concern for me	3.39	1.87
	Friend and Family Support (α = 0.903)		
FSS1	My family really tries to help me.	5.66	1.36
FSS2	I get the emotional help and support I need from my family.	5.75	1.35
FSS3	My friends really try to help me.	5.24	1.41
FSS4	I can count on my friends when things go wrong.	5.15	1.53
FSS5	I can talk about my problems with my family.	5.58	1.41
FSS6	I have friends with whom I can share my joys and sorrows.	5.49	1.41
FSS7	My family is willing to help me make decisions.	5.55	1.45
FSS8	I can talk about my problems with my friends.	5.29	1.39
	Psychological Empowerment (α = 0.916)		
PCE1	The work I do is very important to me.	6.02	0.95
PCE2	My job activities are personally meaningful to me.	5.92	1.06
PCE3	The work I do is meaningful to me.	5.92	1.00
PCE4	I am confident about my ability to do my job.	5.98	1.00
PCE5	I am self-assured about my capabilities to perform my work activities.	5.92	1.02
PCE6	I have mastered the skills necessary for my job.	5.69	1.06
PCE7	I have significant autonomy in determining how I do my job.	5.56	1.07
PCE8	I can decide on my own how to go about doing my work.	5.65	1.15
PCE9	I have considerable opportunity for independence and freedom in how I do my job.	5.61	1.10
PCE10	My impact on what happens in my department is large.	5.06	1.40
PCE11	I have a great deal of control over what happens in my department.	4.55	1.57
PCE12	I have significant influence over what happens in my department.	4.59	1.59
	Facilitating Conditions (α = 0.858)		
FAC1	I have the resources necessary to work from home effectively.	5.43	1.38
FAC2	I have the knowledge necessary to work from home effectively.	5.67	1.22
FAC3	The technology platform provided by the organization is compatible with the work I do from home.	5.49	1.41
FAC4	A specific person (or group) is available for assistance when I experience difficulty when working from home.	5.05	1.68

Note

⁎ reversed coded item

**Table 3:** Mean, standard deviation, average variance extracted (AVE) and reliability of measured constructs.

	WKE	PCE	FSS	RES	FAC	SST
Mean	4.442	5.539	5.464	4.267	5.409	4.772
Std. Deviation	0.910	0.852	1.098	1.186	1.200	1.026
AVE	0.631	0.555	0.595	0.455	0.717	0.707
Cronbach's Alpha	0.924	0.916	0.903	0.757	0.858	0.636
Composite Reliability	0.938	0.936	0.922	0.832	0.910	0.838

**Table 4:** Model summary.

					Change Statistics				
		*R*	Adjusted	Std. Error of	*R* Square	*F*			Sig. *F*
Model	*R*	Square	*R* Square	the Estimate	Change	Change	df1	df2	Change
1	.681^a^	.464	.453	.67288	.464	43.799	5	253	0.001

a Predictors: (Constant), SST, RES, FSS, FAC, PCE

**Table 5:** ANOVA.^a^

Model		Sum of Squares	df	Mean Square	*F*	Sig.
1	Regression	99.153	5	19.831	43.799	0.001^b^
	Residual	114.549	253	.453		
	Total	213.702	258			

a Dependent Variable: WKE

b Predictors: (Constant), SST, RES, FSS, FAC, PCE

**Table 6:** Coefficients.

		Unstandardized Coefficients		Standardized Coefficients		
Model		Beta	Std. Error	Beta	*t*	Sig.
1	(Constant)	.264	.300		0.879	0.380
	PCE	.510	.064	.478	8.003	0.001
	FSS	.075	.046	.091	1.631	0.104
	RES	.160	.040	.208	3.986	0.001
	FAC	.101	.043	.133	2.328	0.021
	SST	-.060	.054	-.068	-1.119	0.264

^a^ Dependent Variable: WKE

The Questionnaire and raw data are attached to the article as supplemental files. The survey was purposefully distributed via Google Form to knowledge workers in the higher educational, engineering / IT services sector following cross-sectional design.

## 2 Experimental Design, Materials, and Methods

The online survey was conducted from April 1 to May 30, 2020, during Malaysia's first movement control order to curb the spread of the COVID-19 pandemic. Except for the essential services, all other business premises were closed during this period, making the online platform the most suitable data collection method. The survey was deployed using the Google form, and the link was shared with the potential respondents via email and social media platforms like Facebook and LinkedIn. We included a cover letter in the survey stating the study's objective and soliciting respondents’ voluntary participation with a promise to keep their responses anonymous. Due to the lack of a sampling frame, we employed the purposive sampling technique by selecting knowledge workers as respondents [1,2]. These respondents were recruited through the authors’ professional networks. Beside, the respondents were requested to assist in sharing the link with their colleagues. The questionnaire design was based on past research, and adaptations were made where necessary. A total of 259 responses were collected.

The survey consists of seven groups of variables, including; (1) demographic data, (2) nine items measurement scale for work engagement, (3) six items measurement scale for resilience, (4) four items measurement scale for supervisor support, (5) eight items measurement scale for family and friend support, (6) 12 items measurement scale for psychological empowerment, and (7) four items measurement scale for facilitating conditions.

Work engagement items were adapted from Schaufeli et al. [3] with Cronbach's alpha, composite reliability and average variance extracted (AVE) values of 0.924, 0.938 and 0.631, respectively. The respondents were asked to respond to nine questions measuring their level of work engagement, based on a seven-point Likert scale ranging from “0”- never to “6” – always.

Resilience items were adapted from Smith et al. [4] with Cronbach's alpha, composite reliability and AVE values of 0.757, 0.832 and 0.455, respectively. The level of respondents agreement to six questions on resilience was assessed using a seven-point Likert scale ranging from “1” - strongly disagree to “7” - strongly agree.

Four items were adapted from Rhoades et al. [5] to measure supervisor support. The respondents were asked to respond to six questions measuring their perception of supervisor support based on a seven-point Likert scale ranging from “1” - strongly disagree to “7” - strongly agree. The Cronbach's alpha, composite reliability and AVE values were 0.636, 0.838 and 0.707, respectively.

Family and friend support items were adapted from Zimet et al. [6] with Cronbach's alpha, composite reliability and AVE values were 0.903, 0.922 and 0.595, respectively. Respondents were asked to assess their level of agreement to questions on Family and friend support, based on a seven-point Likert scale ranging from “1” - strongly disagree to “7” - strongly agree.

Psychological empowerment items were adapted from Spreitzer [7], with Cronbach's alpha, composite reliability and AVE values of 0.916, 0.936 and 0.555, respectively. Respondents were required to respond to 12 questions measuring their level of perceived psychological empowerment using a seven-point Likert scale ranging from “1” - strongly disagree to “7” - strongly agree.

Facilitating conditions items were adapted from Venkatesh et al. [8] with Cronbach's alpha, composite reliability and AVE values of 0.858, 0.910 and 0.717, respectively. Four items were used in assessing respondents’ level of arrangement to questions on facilitating, based on a seven-point Likert scale ranging from “1” - strongly disagree to “7” - strongly agree.

The Cronbach alpha values and composite reliability for some of the variables are considered high (i.e., > 0.90), but studies have suggested that values below 0.95 are desirable [9,10]. Thus, the reported values ranging from 0.636 to 0.938 are considered acceptable. Moreover, Table 7 summarises the results of discriminant validity.

**Table 7:** Result of discriminant validity (Fornell and Larcker Criterion).

Variables	PCE	FAC	FSS	RES	SST	WKE
PCE	**0.754**					
FAC	0.431	**0.847**				
FSS	0.417	0.439	**0.771**			
RES	0.500	0.319	0.328	**0.674**		
SST	0.484	0.561	0.445	0.227	**0.841**	
WKE	0.648	0.412	0.386	0.507	0.364	**0.794**

## Ethics Statement

Since this was a non-experimental, voluntary survey, no ethical approval was required. Informed consent was obtained from participants.

## Declaration of Competing Interest

The authors declare that they have no known competing financial interests or personal relationships, which have, or could be perceived to have, influenced the work reported in this article.

## Appendix Supplementary materials

Image, application 1

Image, application 2
