# Supplementary material for: Survey data on the social, personal, and work resources associated with work engagement among knowledge workers in Malaysia amid the COVID-19 pandemic
Source: Data Brief. 2021 Dec 8;40:107690. doi: 10.1016/j.dib.2021.107690 (PMC8653410; doi:10.1016/j.dib.2021.107690)
Supplement: Supplementary file 2 [file mmc2.docx]

Questionnaire

| **Work Engagement:**  The following statements are about how you feel while working from home due to the outbreak of COVID-19. If you have never had this feeling, tick the “0” (zero) in the space after the statement. If you have had this feeling, indicate how often you felt it by ticking the number (from 1 to 6) that best describes how frequently you feel that way. | | | | | | | | |
| --- | --- | --- | --- | --- | --- | --- | --- | --- |
|  | | **Never** | **Almost Never** | **Rarely** | **Some-times** | **Often** | **Very Often** | **Always** |
| WE1 | At my work, I feel bursting with energy. | 0 | 1 | 2 | 3 | 4 | 5 | 6 |
| WE2 | At my job, I feel strong and vigorous. | 0 | 1 | 2 | 3 | 4 | 5 | 6 |
| WE3 | I am enthusiastic about my job. | 0 | 1 | 2 | 3 | 4 | 5 | 6 |
| WE4 | My job inspires me. | 0 | 1 | 2 | 3 | 4 | 5 | 6 |
| WE5 | When I get up in the morning, I feel like doing my work. | 0 | 1 | 2 | 3 | 4 | 5 | 6 |
| WE6 | I feel happy when I am working intensely. | 0 | 1 | 2 | 3 | 4 | 5 | 6 |
| WE7 | I am proud of the work that I do. | 0 | 1 | 2 | 3 | 4 | 5 | 6 |
| WE8 | I am immersed in my work. | 0 | 1 | 2 | 3 | 4 | 5 | 6 |
| WE9 | I get carried away when I am working. | 0 | 1 | 2 | 3 | 4 | 5 | 6 |
| **Resilience:**  To what extent do you agree or disagree with the following statements on how you have responded to stressful or unexpected situations like the current COVID-19 pandemic. | | | | | | | | |
|  |  | **Strongly Disagree** |  |  |  |  |  | **Strongly Agree** |
| RE1 | I tend to bounce back quickly after hard times. | 1 | 2 | 3 | 4 | 5 | 6 | 7 |
| RE2 | I have a hard time making it through stressful events. | 1 | 2 | 3 | 4 | 5 | 6 | 7 |
| RE3 | It does not take me long to recover from a stressful event. | 1 | 2 | 3 | 4 | 5 | 6 | 7 |
| RE4 | It is hard for me to quickly return back to normal when something bad happens. | 1 | 2 | 3 | 4 | 5 | 6 | 7 |
| RE5 | I usually come through difficult times with little trouble. | 1 | 2 | 3 | 4 | 5 | 6 | 7 |
| RE6 | I tend to take a long time to get over set-backs in my life. | 1 | 2 | 3 | 4 | 5 | 6 | 7 |

| **Social Support:**  The following statements examine your relationships with your work supervisor, family and friends. Please indicate the extent to which you agree or disagree with each statement, based on 7-point Likert scale [i.e. (1) = strongly disagree; (2) = disagree; (3) = somewhat disagree; (4) = neither disagree or agree; (5) = somewhat agree; (6) = agree (7) = strongly agree]. | | | | | | | | |
| --- | --- | --- | --- | --- | --- | --- | --- | --- |
|  | | **Strongly Disagree** |  |  |  |  |  | **Strongly Agree** |
| SS1 | My family really tries to help me. | 1 | 2 | 3 | 4 | 5 | 6 | 7 |
| SS2 | I get the emotional help and support I need from my family. | 1 | 2 | 3 | 4 | 5 | 6 | 7 |
| SS3 | My friends really try to help me. | 1 | 2 | 3 | 4 | 5 | 6 | 7 |
| SS4 | I can count on my friends when things go wrong. | 1 | 2 | 3 | 4 | 5 | 6 | 7 |
| SS5 | I can talk about my problems with my family. | 1 | 2 | 3 | 4 | 5 | 6 | 7 |
| SS6 | I have friends with whom I can share my joys and sorrows. | 1 | 2 | 3 | 4 | 5 | 6 | 7 |
| SS7 | My family is willing to help me make decisions. | 1 | 2 | 3 | 4 | 5 | 6 | 7 |
| SS8 | I can talk about my problems with my friends. | 1 | 2 | 3 | 4 | 5 | 6 | 7 |
| **Facilitating Condition:**  To what extent do you agree or disagree with the following statements on the availability of resources/technologies to support you while working from home since the outbreak of COVID-19 pandemic. | | | | | | | | |
|  |  | **Strongly Disagree** |  |  |  |  |  | **Strongly Agree** |
| FC1 | I have the resources necessary to work from home effectively. | 1 | 2 | 3 | 4 | 5 | 6 | 7 |
| FC2 | I have the knowledge necessary to work from home effectively. | 1 | 2 | 3 | 4 | 5 | 6 | 7 |
| FC3 | The technology platform provided by the organisation is compatible with the work I do from home. | 1 | 2 | 3 | 4 | 5 | 6 | 7 |
| FC4 | A specific person (or group) is available for assistance when I experience difficult when working from home. | 1 | 2 | 3 | 4 | 5 | 6 | 7 |

| **Employee Empowerment:**  To what extent do you agree or disagree with the following statements on how you feel about your ability to shape and influence your work role and context. | | | | | | | | |
| --- | --- | --- | --- | --- | --- | --- | --- | --- |
|  | | **Strongly Disagree** |  |  |  |  |  | **Strongly Agree** |
| EP1 | The work I do is very important to me. | 1 | 2 | 3 | 4 | 5 | 6 | 7 |
| EP2 | My job activities are personally meaningful to me. | 1 | 2 | 3 | 4 | 5 | 6 | 7 |
| EP3 | The work I do is meaningful to me. | 1 | 2 | 3 | 4 | 5 | 6 | 7 |
| EP4 | I am confident about my ability to do my job. | 1 | 2 | 3 | 4 | 5 | 6 | 7 |
| EP5 | I am self-assured about my capabilities to perform my work activities. | 1 | 2 | 3 | 4 | 5 | 6 | 7 |
| EP6 | I have mastered the skills necessary for my job. | 1 | 2 | 3 | 4 | 5 | 6 | 7 |
| EP7 | I have significant autonomy in determining how I do my job. | 1 | 2 | 3 | 4 | 5 | 6 | 7 |
| EP8 | I can decide on my own how to go about doing my work. | 1 | 2 | 3 | 4 | 5 | 6 | 7 |
| EP9 | I have considerable opportunity for independence and freedom in how I do my job. | 1 | 2 | 3 | 4 | 5 | 6 | 7 |
| EP10 | My impact on what happens in my department is large. | 1 | 2 | 3 | 4 | 5 | 6 | 7 |
| EP11 | I have a great deal of control over what happens in my department. | 1 | 2 | 3 | 4 | 5 | 6 | 7 |
| EP12 | I have significant influence over what happens in my department. | 1 | 2 | 3 | 4 | 5 | 6 | 7 |
| **Supervisor Support:**  To what extent do you agree or disagree with the following statements on your supervisor’s concern for your well-being and positive valuation of your contributions to the organisation. | | | | | | | | |
|  |  | **Strongly Disagree** |  |  |  |  |  | **Strongly Agree** |
| SP1 | My supervisor cares about my opinions | 1 | 2 | 3 | 4 | 5 | 6 | 7 |
| SP2 | My work supervisor really cares about my well-being. | 1 | 2 | 3 | 4 | 5 | 6 | 7 |
| SP3 | My supervisor strongly considers my goals and values | 1 | 2 | 3 | 4 | 5 | 6 | 7 |
| SP4 | My supervisor shows very little concern for me | 1 | 2 | 3 | 4 | 5 | 6 | 7 |
